# Comparative Metabolomic Analysis of Four Fabaceae and Relationship to In Vitro Nematicidal Activity against *Xiphinema index*

**DOI:** 10.3390/molecules27103052

**Published:** 2022-05-10

**Authors:** Lise Negrel, Raymonde Baltenweck, Gerard Demangeat, Françoise Le Bohec-Dorner, Camille Rustenholz, Amandine Velt, Claude Gertz, Eva Bieler, Markus Dürrenberger, Pascale Gombault, Philippe Hugueney, Olivier Lemaire

**Affiliations:** 1 Multifolia, 10408 Viâpres-le-Petit, France; contact@multifolia.fr; 2 Santé de la Vigne et Qualité du Vin, INRAE, Université de Strasbourg, 68000 Colmar, France; raymonde.baltenweck@inrae.fr (R.B.); gerard.demangeat@inrae.fr (G.D.); francoise.le-bohec-dorner@inrae.fr (F.L.B.-D.); camille.rustenholz@inrae.fr (C.R.); amandine.velt@inrae.fr (A.V.); claude.gertz@inrae.fr (C.G.); philippe.hugueney@inrae.fr (P.H.); olivier.lemaire@inrae.fr (O.L.); 3 Nano Imaging Laboratory, Swiss Nanoscience Institute, University of Basel, Klingelbergstrasse 50/70, CH-4056 Basel, Switzerland; evi.bieler@unibas.ch (E.B.); markus.duerrenberger@unibas.ch (M.D.)

**Keywords:** *Fabaceae*, *Onobrychis*, *Lotus*, *Melilotus*, *Trifolium*, *Xiphinema index*, grapevine fanleaf degeneration, nematode, nematicide, metabolomics

## Abstract

The grapevine fanleaf virus (GFLV), responsible for fanleaf degeneration, is spread in vineyards by the soil nematode *Xiphinema index*. Nematicide molecules were used to limit the spread of the disease until they were banned due to negative environmental impacts. Therefore, there is a growing interest in alternative methods, including plant-derived products with antagonistic effects to *X. index*. In this work, we evaluated the nematicidal potential of the aerial parts and roots of four *Fabaceae*: sainfoin (*Onobrychis viciifolia)*, birdsfoot trefoil (*Lotus corniculatus)*, sweet clover (*Melilotus albus)*, and red clover (*Trifolium pratense)*, as well as that of sainfoin-based commercial pellets. For all tested plants, either aerial or root parts, or both of them, exhibited a nematicidal effect on *X. index* in vitro, pellets being as effective as freshly harvested plants. Comparative metabolomic analyses did not reveal molecules or molecule families specifically associated with antagonistic properties toward *X. index*, suggesting that the nematicidal effect is the result of a combination of different molecules rather than associated with a single compound. Finally, scanning electron microscope observations did not reveal the visible impact of *O. viciifolia* extract on *X. index* cuticle, suggesting that alteration of the cuticle may not be the primary cause of their nematicidal effect.

## 1. Introduction

Grapevine fanleaf virus (GFLV) is a widely distributed nepovirus responsible for grapevine (*Vitis* spp.) fanleaf degeneration disease [1,2]. Fanleaf degeneration is considered one of the most damaging viral diseases of grapevine, causing serious economic losses by reducing grape yield by up to 80%, affecting fruit quality and shortening the longevity of grapevines with the death of young plants or a progressive decline over many years [3,4]. GFLV is specifically transmitted both via propagating material and by the longidorid ectoparasitic nematode vector *Xiphinema index* using a non-circulative semi-persistent mode [2,5]. *X. index* is widespread in most grapevine growing regions worldwide, often impeding vine cultivation when populations of viruliferous nematodes harboring pathogenic isolates of GFLV are present. *X. index* is extremely resistant and is able to survive at least 4 years without a host plant, in the vineyard or in soil stored between 7 and 20 °C. In addition, the nematodes remain carriers of the virus during this period. Studies have shown that the virus may still be present in the soil after a five-year fallow in an infested vineyard [2,5,6]. *X. index* acquires and transmits virus particles during the feeding process when the nematode inserts its long, retractable hollow stylet into the area of elongation of actively growing rootlets [7]. A gall is formed at the feeding site and it becomes attractive to more individuals and offspring [8,9], causing the spread of the virus in the nematode population and vineyard.

In the absence of GFLV-resistant rootstocks, direct control of vector populations was the main strategy to fight against fanleaf disease, associating cultural practices and soil disinfection with chemical treatments [7,10]. Due to their acute toxicity, most of the synthetic nematicides have been banned or largely restricted in several countries [11,12]. The only methods currently available to control fanleaf disease are the use of certified plant material free from detrimental grapevine viruses including GFLV and the uprooting of the infected vines after devitalization, followed by a long fallow period of over 8 years. However, these approaches are economically unbearable in most vineyards [13].

Alternatives to chemical treatments have been developed, such as hybrid *X. index* tolerant rootstocks (*V. vinifera* × *Muscadinia rotundifolia*) such as the *Nemadex AB* cultivar [14] and transgenic rootstocks expressing sequences from the coat protein (CP) gene of GFLV [15,16]. Jelly and coworkers (2012) [17] developed artificial microRNAs targeting the CP gene of GFLV in grapevine embryos. Currently, the trial has not resulted in an evaluation of the resistance to GFLV and the technique has not been transferred to grapevine plants. Using another biotechnology approach, Hemmer and coworkers (2018) [18] identified a nanobody, a single-domain antigen-binding fragments of camelid-derived heavy-chain-only antibodies (nanobody), with a high affinity to GFLV. Transgenic expression of this nanobody fused to the green fluorescent protein (Chromobody^TM^) in grapevine rootstock confers strong resistance to a broad range of GFLV. However, the negative perceptions towards the use of genetically modified (GM) crops limit the development of GM-derived resistant material [18].

Another approach is soil remediation using cover plants with antagonistic effects, in order to limit the accumulation of the nematode vector. Most identified plant species with nematicide properties belong to the Asteraceae, Fabaceae and Lamiaceae [19]. Experiments in the greenhouse and infected vineyards have shown that some ground cover plants, particularly in the Fabaceae family, have an antagonistic effect against nematodes [20,21]. Indeed, Osei and coworkers (2010) have shown an antagonist effect of *Mucuna pruriens* (Fabaceae), *Crotalaria spectabilitis* (Fabaceae) and *Crotalaria retula* (Fabaceae) against three root-knot nematodes [20]. *Meloidogyne arenaria*, *M. incognita* and *M. javanica* populations failed to multiply in the presence of these plants, no root-gall was induced, and juvenile larva died when they were directly exposed to root or aerial parts extracts. Further work on *Dolichos lablab* (Fabaceae) showed that leaf extracts exhibited nematicidal activity, whereas root extract did not. Finally, Villate and coworkers (2012) [21] studied the effectiveness of different fallow plants on *X. index* multiplication rate. Fabaceae appear to be predominantly nematicidal. This work has highlighted the antagonist effect of *Lotus corniculatus* (birdfoot trefoil), *Lupinus albus* (white lupine), *Trifolium pratense* (purple clover), *Onobrychis viciifolia* (sainfoin), *Medicago hybrid* (alfalfa) and *Vicia vilosa* (hairy vetch). Conversely, *Crotalaria spectabilis* (rattlesnake) and *Melilotus albus* (white melilot) had no impact on *X. index* populations. Molecules present in some of these Fabaceae have already been explored [22,23,24,25], but none of these have been identified as a nematicide.

Spreading or burying nematicide plant materials could also be an alternative to the use of synthetic nematicides. Indeed, pellets of sainfoin have shown some benefits on livestock health, due to their anthelmintic potential. Such pellets have been shown to decrease meteorization, improve cattle digestion, and increase the levels of polyunsaturated fatty acids in lamb meat [26,27]. Pellets also have a nematicidal effect by reducing the fertility of *Haemonchus contortus* [28], and limiting the development of *Trichostrongylus colubrifornis* eggs in rabbits’ feces [29]. In calves fed with sainfoin pellets, infestations of *Ostertagia ostertagi* were reduced [30]. Pellets are also anti-eimerial and reduce the excretion of *Eimeria* oocyst in rabbits [31]. The successful use of plant-derived products with anthelmintic properties to promote animal health has prompted us to evaluate the potential of such products in plant health, to fight a virus-vectoring plant nematode.

In this work, we evaluated the nematicidal potential against *X. index* of the aerial parts and roots of four Fabaceae: sainfoin (*Onobrychis viciifolia)*, birdsfoot trefoil (*Lotus corniculatus)*, sweet clover (*Melilotus albus)*, and red clover (*Trifolium pratense)*, as well as that of sainfoin-based commercial pellets. In vitro survival assays using *X. index* showed that some of the tested Fabaceae exhibited a significant nematicidal effect, pellets being as effective as freshly harvested plants. Comparative metabolomic analyses using Ultra-High Performance Liquid Chromatography coupled to Mass Spectrometer (UHPLC-MS) did not reveal molecules or molecule families specifically associated with antagonistic properties toward *X. index*, suggesting that the nematicidal effect is the result of a combination of different molecules.

## 2. Results

### 2.1. In Vitro Antagonistic Effect against X. index of the Aerial and Root Parts of Different Fabaceae

In order to evaluate potential antagonistic effects toward *X. index*, extracts of aerial parts (A) and roots (R) of birdsfoot trefoil (BT), sweet clover (SC), red clover (RC) and sainfoin (SF) were prepared from 11-week-old plants, as well as an extract of commercial sainfoin-based pellets (SF-P). Such pellets are made from aerial parts of 18-month-old sainfoin plants. Fifty nematodes were incubated in these extracts at various concentrations (0, 5, 10, 20 g·L^−1^) and monitored for 72 h to determine nematode mortality. Dead nematodes are inactive and typically comma-shaped [32]. Unaffected nematodes are active and present flexible shapes. Attesting death requires the transfer of immobile nematodes into water. If the nematode regains mobility, the effect is reversible and the extracts are qualified as nematostatic. Using these bioassays, all immobile nematodes in Fabaceae aqueous extracts did not recover after transfer in water for 24 h. Fabaceae extracts were therefore qualified as nematicide rather than nematostatic in these conditions. *X. index* are able to survive in water for one to two weeks. In this bioassay, the mortality of nematodes was 7.28% on average after three days of incubation in controlled wells containing 1% PBS.

**Figure 1:**
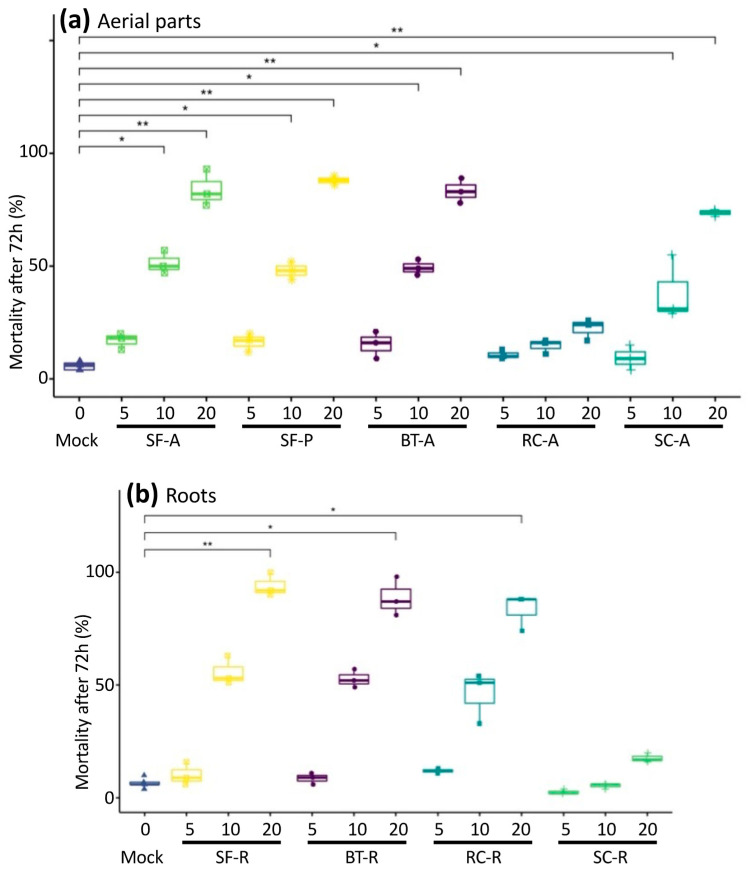
In vitro antagonistic effect of Fabaceae extracts on *X. index*. Nematodes were exposed to control conditions (1% PBS = Mock) or to the indicated Fabaceae extracts at 5, 10 and 20 g·L^−1^ for 72 h. (**a**) Aerial parts extracts; (**b**) Root extracts. Impact of Fabaceae extracts on *X. index* was assessed by measuring mortality rate in %. Three independent repetitions of the experiment were performed with *n* = 50 *X. index* for each condition. * and ** indicate significantly different mortality compared to the Mock condition using a Dunn test, with adjusted *p*-value < 0.05 and <0.01, respectively. SF: sainfoin, BT: birdsfoot trefoil, RC: red clover, SC: sweet clover, A: aerial parts, R: roots, P: pellets.

Most aerial part extracts at 10 g·L^−1^ and 20 g·L^−1^ induced high mortality of *X. index* except for RC-A extracts, whose nematicide effect on *X. index* was minor (Figure 1a). The mortality rates of the 20 g·L^−1^ extracts after 72 h reached 89%, 91% 67% and 22% for SF-A, BT-A, SC-A and RC-A extracts, respectively. SF-P extract was as effective as the aerial parts of SF, with 87% mortality at 20 g·L^−1^ after 72 h. After 24 h exposure to plant extracts, the mortality of nematodes was not significantly different from that observed in mock wells, regardless of the plants used and the concentrations tested. The 50% mortality rate (MR_50_) was reached after 72 h in extracts of SF and BT at 10 g·L^−1^ and 48 h in solutions at 20 g·L^−1^. The MR_50_ was not reached before 72 h at 20 g·L^−1^ in the SC-A extracts (Supplementary Figure S1).

Root extracts at 10 and 20 g·L^−1^ showed a strong nematicidal effect, except for sweet clover. After 72 h, the mortality rates of the 20 g·L^−1^ solutions reached 95%, 90% 18% and 83% for SF-R, BT-R, SC-R and RC-R extracts, respectively (Figure 1b). The mortality in solutions at 20 g·L^−1^ could be observed from 24 h, except for RC-R, whose mortality was only significant from 48 h. However, the MR_50_ was exceeded after 48 h in SF-R and BT-R extracts concentrated at 10 g·L^−1^ and 20 g·L^−1^, while an incubation for 72 h at a concentration of 20 g·L^−1^ was necessary for RC-R extracts (Supplementary Figure S1). The extracts concentrated at 5 g·L^−1^ did not show any nematicidal effect, in these conditions and extracts at 10 g·L^−1^ were not significantly nematicidal before 48 h.

Altogether, six of nine Fabaceae extracts tested here significantly affected the in vitro survival of *X. index* (Figure 1), with time and dose effects. Sainfoin cv. Perly and lotier cv. Altus were particularly interesting because of the whole plant effect and nematicidal efficiency against *X. index*. Conversely only roots of red clover cv. Formica and aerial parts of sweet clover showed significant antagonist effect against *X. index*. For all nematicidal plant extracts, mortality in 10 and 20 g·L^−1^ solutions increased with time without reaching a plateau, indicating that the nematode mortality rate could increase further with longer exposure times (Supplementary Figure S1). As all the plants tested in this study belong to the Fabaceae family, their metabolome may share some similarities. Therefore, a metabolomic analysis was carried out in order to highlight compounds or molecule families potentially associated with the in vitro nematicidal effect.

### 2.2. Targeted Comparative Metabolomic Analyses of Fabaceae Extracts

To gain further insights into the molecular bases of the nematicidal effect of the selected *Fabaceae*, a metabolomic analysis was performed using UHPLC-MS. Metabolites chosen as targets were selected based on previously published analyses of plants in the Fabaceae family, particularly in relationship with anthelmintic properties [22,23,24,25,33,34]. As many of these compounds belong to polyphenols and especially flavonoids, roots and aerial parts of the selected Fabaceae were extracted with methanol:water (1:2, *v*/*v*) as a solvent, in order to enhance the extraction of such semi-polar metabolites. In addition to previously identified metabolites [22,23,24,25,33,34], a number of compounds present in high amounts in the selected extracts were identified, based on expertized analysis of mass spectra and comparison with published literature. Using these criteria, 93 metabolites were selected for relative quantification in all Fabaceae extracts (Supplementary Table S1). Out of these 93 potential targets, 82 metabolites were detected in quantifiable amounts in at least one of the plant extracts analyzed. These 82 targeted molecules included 14 cinnamic acid and derivatives (CA), 40 flavonoids (F), 8 organic acids (OA), 20 proanthocyanidins and flavanols (PF). Based on the retention time and available standards, procyanidins were identified as procyanidin A2, B1, B2 and C2. Based on accurate mass, fragmentation analysis and retention times, 12 different prodelphinidin oligomers were tentatively identified, including 5 dimers, 6 trimers and one tetramer. Putative prodelphinidin dimers 1–5 corresponded to compounds with molecular formula C_30_H_26_O_13_ or C_30_H_26_O_14_, with different retention times. Similarly, putative prodelphinidin trimers 1–6 corresponded to compounds with molecular formula C_45_H_38_O_19_, C_45_H_38_O_20_, C_45_H_38_O_21_ and C_45_H_36_O_21_, with different retention times. Finally, a compound with the molecular formula C_60_H_50_O_27_ was tentatively identified as a prodelphinidin tetramer. Detailed characteristics of these prodelphinidin oligomers are given in Supplementary Table S1.

**Figure 2:**
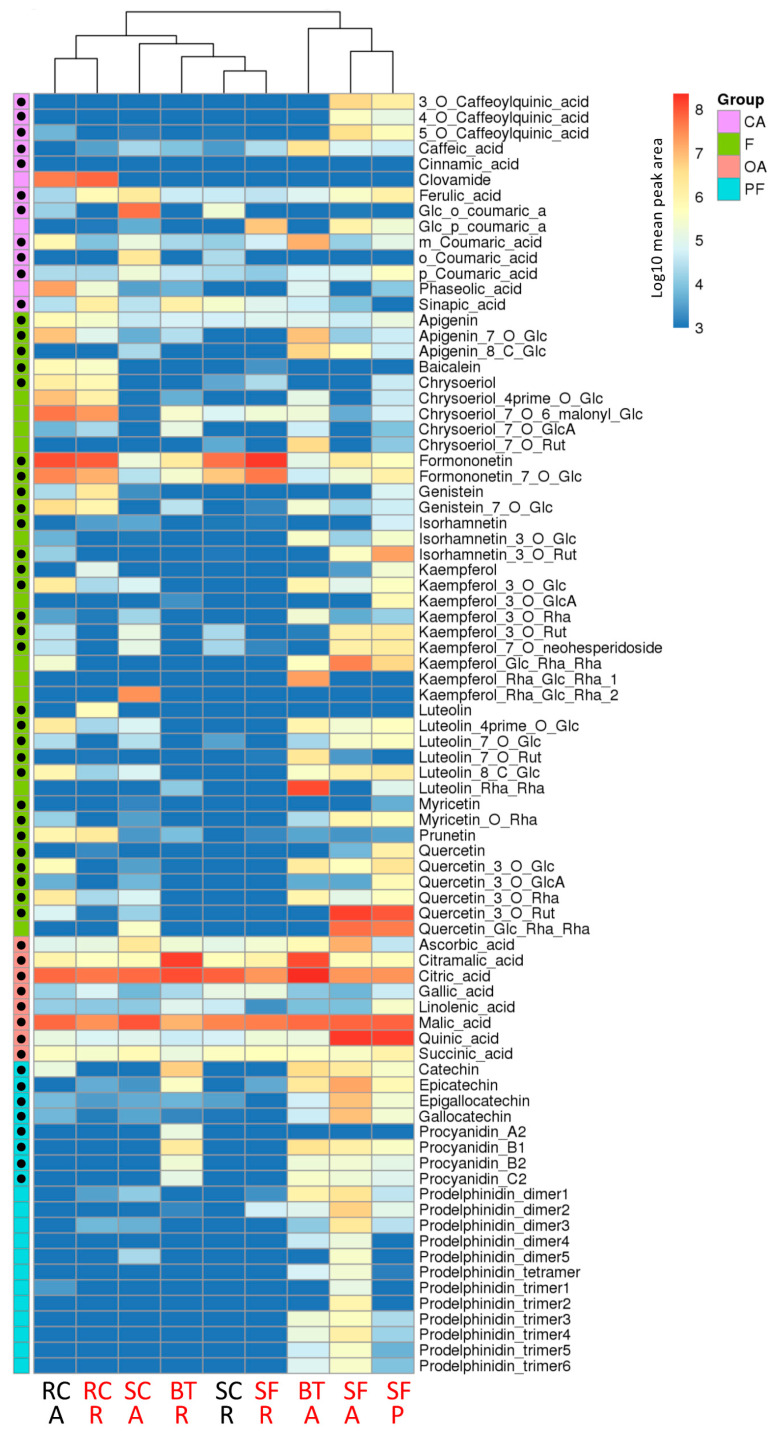
Metabolic profiling of Fabaceae extracts. Log10 mean peak areas of the indicated metabolites are given by shades of red, yellow or blue colors according to the scale bar. Metabolites are grouped as cinnamic acid and derivatives (CA), flavonoids (F), organic acids (OA) and proanthocyanidins and flavanols (PF). Identity of the metabolites indicated with black dots was confirmed with the corresponding standard. SF: sainfoin, BT: birdsfoot trefoil, RC: red clover, SC: sweet clover, A: aerial parts, R: roots, P: pellets. Weakly nematicidal and highly nematicidal extracts are indicated in black and red, respectively. Hierarchical clustering highlights similarity among samples. For glycosides, the following abbreviations were used: glucoside: Glc; glucuronide: GlcA; rhamnoside: Rha; rutinoside: Rut.

**Figure 3:**
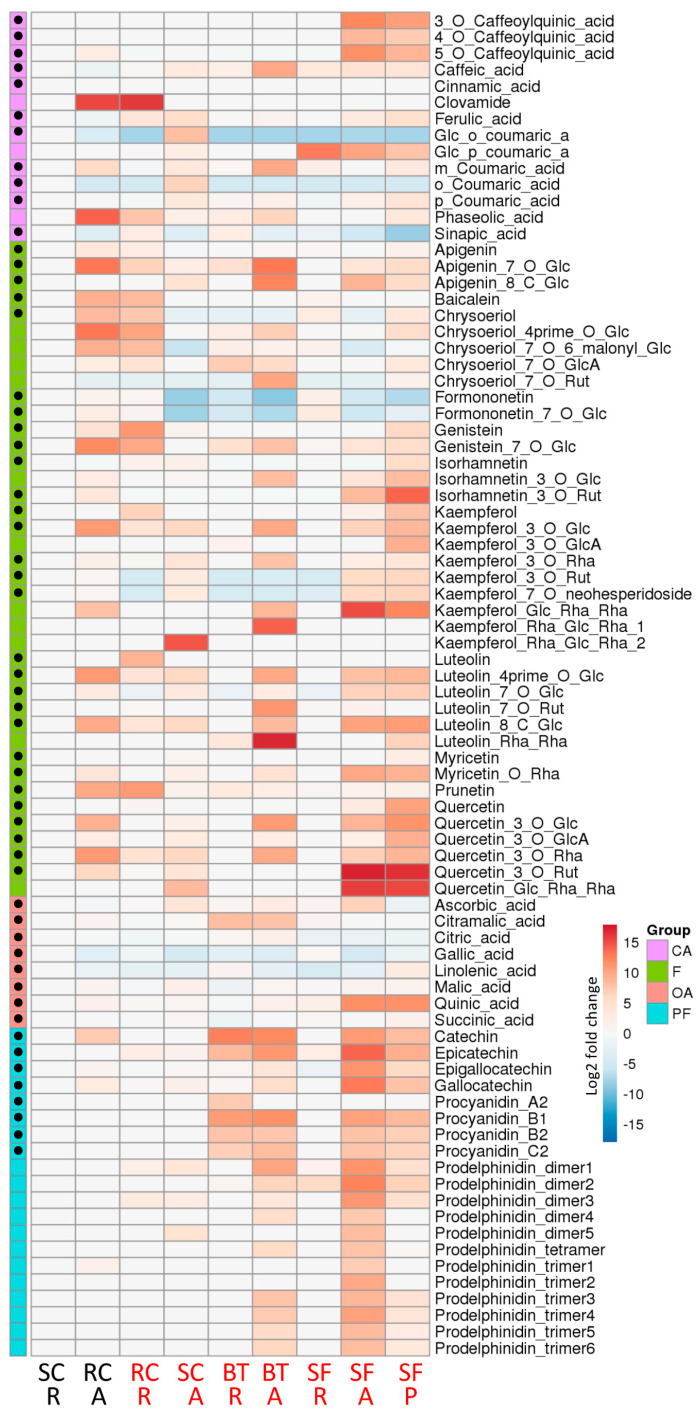
Comparative metabolomic analysis of Fabaceae extracts. Mean relative abundance of the selected metabolites are presented as Log2 ratios compared to the weakly nematicidal root extract of sweet clover (SC-R), with shades of red or blue colors according to the scale bar. Metabolites are grouped as cinnamic acid and derivatives (CA), flavonoids (F), organic acids (OA) and proanthocyanidins and flavanols (PF). Identity of the metabolites indicated with black dots was confirmed with the corresponding standard. SF: sainfoin, BT: birdsfoot trefoil, RC: red clover, SC: sweet clover, A: aerial parts, R: roots, P: pellets. Weakly nematicidal and highly nematicidal extracts are indicated in black and red, respectively. Glucoside: Glc; glucuronide: GlcA; rhamnoside: Rha; rutinoside: Rut.

A principal component analysis (PCA) based on all metabolites was performed to determine whether the targeted metabolites allow differentiation between the Fabaceae samples (Supplementary Figure S2). The first two dimensions explaining, respectively, 26% and 16% of the variance, allowed a good differentiation of SF-A, SF-P, BT-A and BT-R extracts. Conversely, all other extracts were grouped together, independently from their aerial or root origin. Furthermore, no clear separation was observed between highly nematicidal and weakly nematicidal extracts. Metabolic profiling of the Fabaceae extracts revealed distinctive patterns of metabolite accumulation, depending on organs and species (Figure 2). Several compounds were detected in all analyzed samples, especially in the organic acid family. Formononetin derivatives were present in most samples as well. Conversely, other compounds or compound families were restricted to specific sets of samples. For example, significant amounts of caffeoylquinic acids, flavanols and proanthocyanidins were almost exclusively detected in sainfoin-derived extracts. Similarly, red clover extracts were rich in chrysoeriol derivatives. However, hierarchical clustering did not allow to group highly nematicidal or weakly nematicidal extracts together (Figure 2). In order to look further for compounds potentially associated with the nematicidal effect, the metabolite contents of all extracts were compared to that of the weakly nematicidal root extract of sweet clover (SC-R) (Figure 3). Some compounds accumulated to remarkably high levels in some plants, such as rutin (quercetin-3-*O*-rutinoside) in aerial parts or pellets of sainfoin, or luteolin-rhamnosyl-rhamnoside in aerial parts of birdsfoot trefoil. As observed with the PCA (Figure S2) and the hierarchical clustering (Figure 3), metabolite contents of root extract of sweet clover (SC-R) and sainfoin (SF-R) appeared quite similar, although they exhibited very different nematicidal properties. In order to highlight metabolites potentially associated with nematicidal properties, volcano plot analyses were used to compare highly nematicidal (HN) to weakly nematicidal (WN) plant extracts. Comparison of sweet clover extracts SC-A (HN)/SC-R (WN) and red clover extracts RC-R (HN)/RC-A (WN) highlighted metabolites significantly accumulated in HN extracts (Supplementary Figure S3a,b). However, a comparison of all HN to all WN extracts did not allow the identification of candidate nematicidal metabolites, as no metabolite was significantly more accumulated in HN extracts (Figure S3c). Altogether, a comparison of weakly nematicidal and highly nematicidal extracts did not highlight molecules or molecule families specifically associated with nematicidal properties among the targeted metabolites.

### 2.3. Impact of Sainfoin Pellets Extract on the Cuticle of X. index

**Figure 4:**
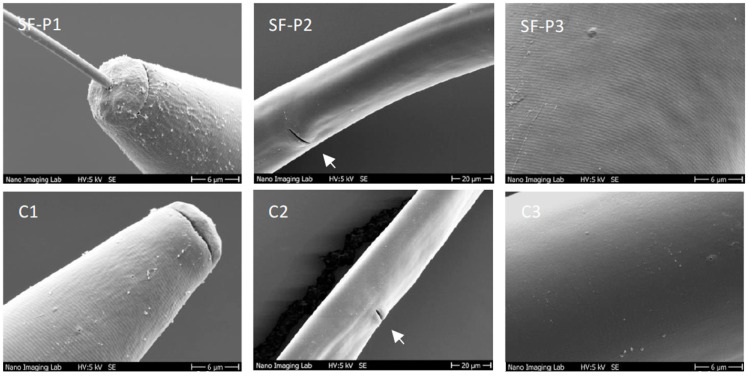
Observation of *X. index* under Scanning Electron Microscope (SEM). Observation of sexual region (arrow indicates vulva) (SF-P1/C1), cephalic region (SF-P2/C2) and cuticle surface (SF-P3/C3) of *X. index*, after treatment with sainfoin pellet extract at 20 g·L^−1^ during 72 h (SF-P1–3) or incubation in control conditions (PBS 1%) (C1–3).

Condensed tannins (proanthocyanidins) have been proposed to alter the external cuticle of gastrointestinal nematodes, thus affecting their life cycle and their viability [35,36]. As sainfoin extracts were rich in proanthocyanidins (Figure 3), sainfoin pellet extracts (SF-P) were evaluated for their potential to alter the cuticle of *X. index*. nematodes were observed under a scanning electron microscope (SEM) after three days of incubation in sainfoin extract at 20 g·L^−1^ (Figure 4, SF-P1, SF-P3), and compared with nematodes incubated in PBS 1% (control; Figure 4, C1–C3).

No major modification of the cuticle was observed in *X. index* incubated with SF-P extract compared to the control. Cuticle surfaces were smooth, apart from patches where it seemed slightly peeled. These patches appeared in both SF-P and control conditions (Figure 4, SP3 vs. C3). No aggregates around the vulva were observed, unlike previously described on the cattle nematode *H. contortus* [35]. Finally, the cuticle of the cephalic region appeared slightly withered, with thicker ridges and aggregates or debris after sainfoin treatment. In the control assays, these aggregates were also observable, the cuticle appearing overall slightly smoother. Overall, sainfoin extracts did not seem to significantly impact the external structure of *X. index* under our experimental conditions, although they induced high mortality. Finally, as precursors of proanthocyanidins, the flavanols gallocatechin and epigallocatechin were tested for potential nematicidal effect on *X. index.* However, no impact of these molecules on *X. index* survival was observed (results not shown).

## 3. Discussion

There is currently high interest in developing environmentally friendly alternatives to synthetic nematicides in agriculture. In this work, we show that extracts of the aerial parts and roots of Fabaceae exhibit interesting nematicidal properties against *X. index*, the vector of the main viral disease affecting viticulture worldwide. Using an in vitro bioassay based on the monitoring of *X. index* viability over 72 h, we evaluated the nematicidal potential of extracts of aerial parts and roots of four Fabaceae. Among them, sainfoin cv. Perly and birdsfoot trefoil cv. Altus appeared particularly efficient, both aerial part and root extracts showing high antagonistic properties to *X. index*. The nematicidal properties of both sainfoin and birdsfoot trefoil extracts exhibited time and dose effects, with nearly 90% *X. index* mortality after 72 h of incubation in the 20 g·L^−1^ extracts. Conversely, some Fabaceae showed contrasted nematicidal properties when comparing the aerial parts and root extracts. Root extracts of sweet clover were weakly nematicidal compared to the aerial parts of the same plant and the opposite was true with red clover extracts. Interestingly, extracts of commercial sainfoin pellets were as effective as extracts of freshly harvested aerial parts of sainfoin.

As expected, metabolomic analyses of root and aerial part extracts revealed metabolic heterogeneity among Fabaceae species, although some Fabaceae extracts presented quite similar profiles of the selected metabolites. This was especially true for root extract of sweet clover (SC-R) and sainfoin (SF-R), despite the fact that they exhibited different nematicidal properties. Although none of the quantified metabolites was exclusively present in nematicidal extracts, some compounds were present in higher amounts in such extracts. For example, epicatechin and procyanidin B1 were more abundant in highly nematicidal aerial part extracts of sainfoin and birdsfoot trefoil. Epicatechin has been shown to be antagonistic to *H. contortus*, with an EC_50_ of 0.01 g·L^−1^ [37]. A nematicidal effect of epicatechin against *Caenorhabditis elegans* and *Onchocerca ochengi* has also been reported previously [38]. The 11-week-old sainfoin aerial parts (SF-A) contained more oligomeric proanthocyanidins than the commercial sainfoin pellets prepared from 18-month-old plants, albeit without affecting the nematicidal properties of the corresponding extracts.

Our comparative analysis of the Fabaceae aerial part showed the presence of the cinnamic acid derivative caffeic acid (CA) in some extracts with antagonistic properties to *X. index*. The antagonistic effect of CA against *X. index* was previously highlighted [39] when studying the nematicidal potential of *Artemisia annua*. CA was also reported to differentially affect the plant nematodes *Radopholus similis*, *Meloidogyne incognita* and *Pratylenchus penetrans* [40]. Indeed, CA showed no impact on *P. penetrans* but was lethal to *R. similis* (LC_50_ at 3.5 g·L^−1^). CA exhibited anthelmintic and ovicidal effects on the cattle nematode *H. contortus* [41,42]. In addition, the nematicidal properties of CA were shown to be influenced by other molecules such as quercetin, suggesting a synergistic interaction between caffeic acid and quercetin [43]. Such synergistic effects have been shown between quercetin, CA, rutin and coumarin, for in vitro anthelmintic activity against the cow nematode *Cooperia punctata* [44]. Interestingly, volcano plot analyses highlighted cinnamic acid derivatives such as coumaric acids and derivatives, ferulic acid, sinapic acid and clovamide as potentially associated with the nematicidal properties of sweet clover and red clover extracts (Supplementary Figure S3a,b). Future work will be needed to investigate the potential involvement of cinnamic acid derivatives in the nematicidal properties of these Fabaceae.

Quercetin and its derivatives have shown antagonistic properties to a number of nematode species, including *M. incognita*, *R. similis*, *P. penetrans*, *H. contortus* [37,40,45]. In addition, rutin isolated from *Jasminum grandiflorum* was identified as a highly anthelmintic compound against the horse nematode *Habronema muscae* with an LC_50_ of 0.04 g·L^−1^/24 h [46]. In human health, quercetin is antagonistic to *Encephalitozoon intestinalis*, with an EC_50_ of 5 × 10^−6^ M [47]. The action of quercetin may be related to the inhibition of ATP-dependent transporters associated with multiple resistance to drugs such as P-glycoprotein, which is the main mechanism associated with resistance to macrocyclic lactones in nematodes [48,49,50]. Quercetin and its derivatives, in particular rutin, were detected in large amounts in the aerial parts and the pellets of sainfoin, and to a lesser extent in extracts of birdsfoot trefoil and red clover. Quercetin and its derivatives may therefore participate in the antagonistic effects of sainfoin and birdsfoot trefoil against *X. index*, probably in association with other compounds promoting synergistic effects. In addition, some metabolites that were not targeted in our metabolomic analyses may participate in the nematicidal properties of the four Fabaceae studies here. Indeed, metabolites chosen for targeted quantification in Fabaceae extracts were selected based on reported antagonistic properties mainly against animal parasitic nematodes. It is likely that plant nematodes such as *X. index* have evolved mechanisms allowing them to cope with an array of plant secondary metabolites, including those present in the host plant. Therefore, some plant metabolites with antagonistic properties to animal parasitic nematodes may not be as efficient against plant nematodes. A volcano plot comparison of all Fabaceae extracts analyzed in this work based on their nematicidal properties (Supplementary Figure S3c) did not highlight metabolites significantly accumulated in nematicidal extracts. Future work will therefore be needed to explore the metabolome of antagonistic Fabaceae, in order to identify new metabolites with nematicidal properties against plant-parasitic nematodes such as *X. index.* Nevertheless, UHPLC-MS analyses performed in this work provide a significant survey of more than 80 compounds present in at least one of the four selected Fabaceae species, the identification of 57 of these compounds being confirmed with authentic standards. This constitutes a valuable basis for future metabolomic analyses of other plants of interest in the Fabaceae family.

In some cases, sainfoin-based products have been shown to physically alter the external structures of animal parasitic nematodes. For example, damages and particle aggregates appeared on the outer cuticle of *Ostertagia ostertagi* from calves fed with sainfoin pellets. These damages have been proposed to be associated with condensed tannins [51]. In addition, polyphenols and especially condensed tannins are suspected of causing internal lesions in *H. contortus*. Indeed, tannin-rich plant extracts have been shown to induce the appearance of numerous vesicles in the cytoplasm, together with the alteration of the muscular and intestinal structures [52,53]. In our work, scanning electron microscope observations did not reveal any visible alteration of *X. index* external structures in the presence of a condensed tannins-rich extract of sainfoin pellets, despite its significant nematicidal activity. Therefore, the impact of condensed tannins may be different on plant and animal parasitic nematodes and the alteration of cuticle may not be the primary cause of the observed nematicidal effect of sainfoin extracts on *X. index*.

## 4. Materials and Methods

### 4.1. Xiphinema index Culture

Aviruliferous populations of *X. index* were reared on fig plants (*Ficus carica*) kept in 10 L containers filled with an artificial substrate composed of loess and sand (1/4:3/4, *w*/*w*) in a greenhouse under controlled temperature (20–25 °C) and light (16 h photoperiod) conditions.

### 4.2. Plant Materials and Plant Extracts

*Onobrychis viciifolia* cv. Perly (sainfoin), *Lotus corniculatus* cv. Altus (birdsfoot trefoil) and *Trifolium pratense* cv. Formica (red clover) seeds were provided by Cerience (Cisse, France). *Melilotus albus* (sweet clover) seeds were produced by Graines Baumaux, S.A.S., FR. Plants were cultivated in the greenhouse for 11 weeks under controlled temperature (20–25 °C) and light (16 h photoperiod) conditions. Commercial Vitifolia^®^ pellets were provided by Multifolia (Viâpres-le-Petit, France). Pellets were prepared from dehydrated aerial parts of 18-months-old *O. viciifolia* cv. Perly. Aqueous extracts were prepared from freeze-dried plant material, either from aerial parts or roots. The dried plant material was powdered by a mill and sieved (mesh at 1 mm). The plant powders (2 g ± 50 mg) were mixed with a 1% phosphate-buffered saline (PBS) solution at a concentration of 200 g·L^−1^, sonicated for 5 min and heated at 80 °C for 25 min. Aqueous solutions were centrifugated at 5000× *g* for 15 min. Supernatants were diluted within a 1% PBS to obtain extracts corresponding to 5, 10 and 20 g·L^−1^.

### 4.3. Nematode Survival Bioassay in Aqueous Medium

Adult nematodes were isolated from the rearing soil by sieving methods as described by Flegg (1967) [54]. Soil samples were filtered through sieves of different meshes using tap water. *X. index* nematodes were identified, washed in 1% PBS and numbered under a binocular microscope. Three lots of 50 isolated nematodes were incubated in the dark at 20 °C in plastic wells (Fisher Scientific SAS, Illkirch-Graffenstaden, France) filled with 2 mL of the plant extract in 1% PBS at 20, 10, 5 or 0 g·L^−1^ concentrations. Nematode viability was recorded after 24, 48 and 72 h under binoculars by scoring their mobility. Non-mobile nematodes were isolated and placed in 1% PBS for 24 h in order to discriminate between nematostatic or lethal effects of the solutions tested.

### 4.4. Metabolite Extraction for Metabolomic Analyses

Aerial parts of the plants, roots and sainfoin pellets were freeze-dried and weighed. After grinding the sample using a bead mill (TissueLyser II, Qiagen, Les Ulis, France), metabolites were extracted according to Castillo-Mitre et al. (2017) [41] with some modifications. Namely, 200 mg of plant material was extracted with MeOH/H_2_O (1/2, *v*/*v*) in order to maximize the extraction of semi-polar metabolites, using 5 μL per mg dry weight. The samples were sonicated for 15 min and centrifugated at 15,000× *g* for 10 min. The supernatant phase was then directly analyzed by UHPLC-MS.

### 4.5. Metabolomic Analyses

Analyses were carried out using a Dionex Ultimate 3000 ultra-high performance liquid chromatography (UHPLC, Thermo Fisher Scientific, Waltham, MA, USA) system, equipped with a Nucleodur C18 HTec column (Macherey-Nagel, Düren, Germany, 150 mm × 2 mm internal diameter, 1.8 μm diameter particles) maintained at 30 °C. Eluents used were as follows: water/formic acid (0.1%, *v*/*v*; eluent A) and acetonitrile/formic acid (0.1%, *v*/*v*; eluent B); the flow rate was maintained at 0.25 mL.minute^−1^. The separation program used was as follows: 80% eluent A under the initial conditions, 80% to 70% eluent A in 4 min, 70% to 50% eluent A in 1 min, 1.5 min isocratic at 50% eluent A, 50% to 1% eluent A in 2 min and finally isocratic with 1% eluent A for 1.5 min. The volume of the sample injected was 1 μL. The chromatographic system was coupled to an exactive high-resolution mass spectrometer (HRMS, Thermo Fischer Scientific, San Jose, California, USA) equipped with an electrospray ionization source operating in positive or negative mode. The spectrometer parameters are set at 300 °C for the temperature of the ion transfer capillary and 3500 V and 2500 V, respectively, in positive and negative mode for the needle voltage. The nitrogen flow rates of the sheath gas and the auxiliary gas are maintained at 40 and 5 (in arbitrary units), respectively. Spectra were acquired within the *m*/*z* mass range between 100 to 1300 u, using a resolution of 50,000 at *m*/*z* 200 u. The system was calibrated externally using a calibration solution (Pierce™ LTQ ESI Negative Ion solution, ThermoFisher Scientific), giving a mass accuracy lower than 5 ppm in the negative mode. In the positive mode, the system was calibrated internally using dibutyl phthalate as lock mass at 279.591 u, giving a mass accuracy lower than 1 ppm. The instruments were controlled by the Xcalibur software and the exact *m*/*z* and retention time of each metabolite were used for targeted metabolomic analyses using the Xcalibur software. The selected metabolites were grouped into four major chemical or functional families, including cinnamic acid and derivatives (CA), flavonoids (F), organic acids (OA), proanthocyanidins and flavanols (PF). For some metabolites, identity was confirmed with the corresponding authentic standard provided by Sigma-Aldrich (Saint-Quentin-Fallavier, France) or Extrasynthese (Genay, France) (Supplementary Table S1).

### 4.6. Microscopic Observations

In order to observe the impact of the aqueous extractions of sainfoin pellets on the external surface of *X. index*, approximately 200 nematodes were incubated in a concentrated extraction at 20 g·L^−1^ and another 100 were incubated in 1% PBS as mock. After 3 days, around twenty weak but still alive nematodes were selected for the pellet extraction. A total of 20 other nematodes were recovered from the 1% PBS solution. For microscopic observations, nematodes were prepared as described previously [35]. In brief, the nematodes were fixed in 4% glutaraldehyde in 0.1 M PBS (pH 7.2) for over 24 h at 4 °C. After washing with ultrapure water and dehydrated in an ascending ethanol series up to 100%, nematodes were critical point dried, mounted and sputtered (coated) with gold. Finally, nematodes were observed (under) with a scanning electron microscope (Philips XL30 ESEM).

### 4.7. Statistical Analysis

Statistical analyses were performed using the R software version 3.5.1 (R Core Team, 2018). All experiments were repeated three times and the mean values of the three experiments were used. Data collected during *X. index* survival bioassays were subjected to a Dunn test at 5% probability, using the R package dunn.test (version 1.3.5). Heatmaps were constructed using the package ComplexHeatmap [55]. Principal component analysis (PCA) was obtained using the package vegan [56].

## 5. Conclusions

In this work, we evaluated the nematicidal potential of the aerial parts and roots of four Fabaceae: sainfoin, birdsfoot trefoil, sweet clover, and red clover, as well as that of sainfoin-based commercial pellets. Using a nematode survival test, we have highlighted the nematicidal effects of some extracts of these Fabaceae against *X. index* in vitro. In addition, UHPLC-MS analyses of the selected Fabaceae extracts have provided a significant survey of more than 80 compounds, constituting a valuable basis for future metabolomic analyses of other plants of interest in the Fabaceae family. Comparative metabolomic analyses did not reveal molecules or molecule families specifically associated with antagonistic properties, suggesting that the nematicidal effect is the result of a combination of different molecules rather than associated with a single compound. In addition, nematicidal properties may come from other metabolites, which still need to be identified. As this study has shed light on the nematicidal and antagonistic properties of Fabaceae plants and extracts, it will be of great interest to analyze their impact on the retention and transmission of nepoviruses. Such a biocontrol strategy might have two benefits, decreasing the *X. index* populations and thus the infectious potential of fanleaf infested soils and limiting the spread of the disease in a vineyard. Therefore, due to their antagonistic properties, Fabaceae-derived extracts or products may constitute interesting alternatives to synthetic nematicides in agriculture, especially with the increasing development of organic farming. As a standardized and easily available resource, sainfoin pellets are currently being used in larger-scale experiments for the evaluation of potential antagonistic properties to *X. index* in the vineyard.

## Data Availability

The data presented in this study are available on request from the corresponding author.

## Supplementary Materials

The following supporting information can be downloaded at: https://www.mdpi.com/article/10.3390/molecules27103052/s1, Figure S1: Effect of aerial part and root extracts of Fabaceae on the mortality of *X. index* in vitro; Table S1: List of the 93 targets for metabolomic analyses; Figure S2: Principal component analysis of metabolite contents in Fabaceae extracts; Figure S3: Volcano plot analyses of differentially accumulated metabolites between highly nematicidal and weakly nematicidal Fabaceae extracts.
