# Supplementary material for: Comparative Metabolomic Analysis of Four Fabaceae and Relationship to In Vitro Nematicidal Activity against Xiphinema index
Source: Molecules. 2022 May 10;27(10):3052. doi: 10.3390/molecules27103052 (PMC9146138; doi:10.3390/molecules27103052)
Supplement: Supplementary file 1 [file molecules-27-03052-s001.zip › molecules-1680582-supplementary.pdf]

Supplementary material

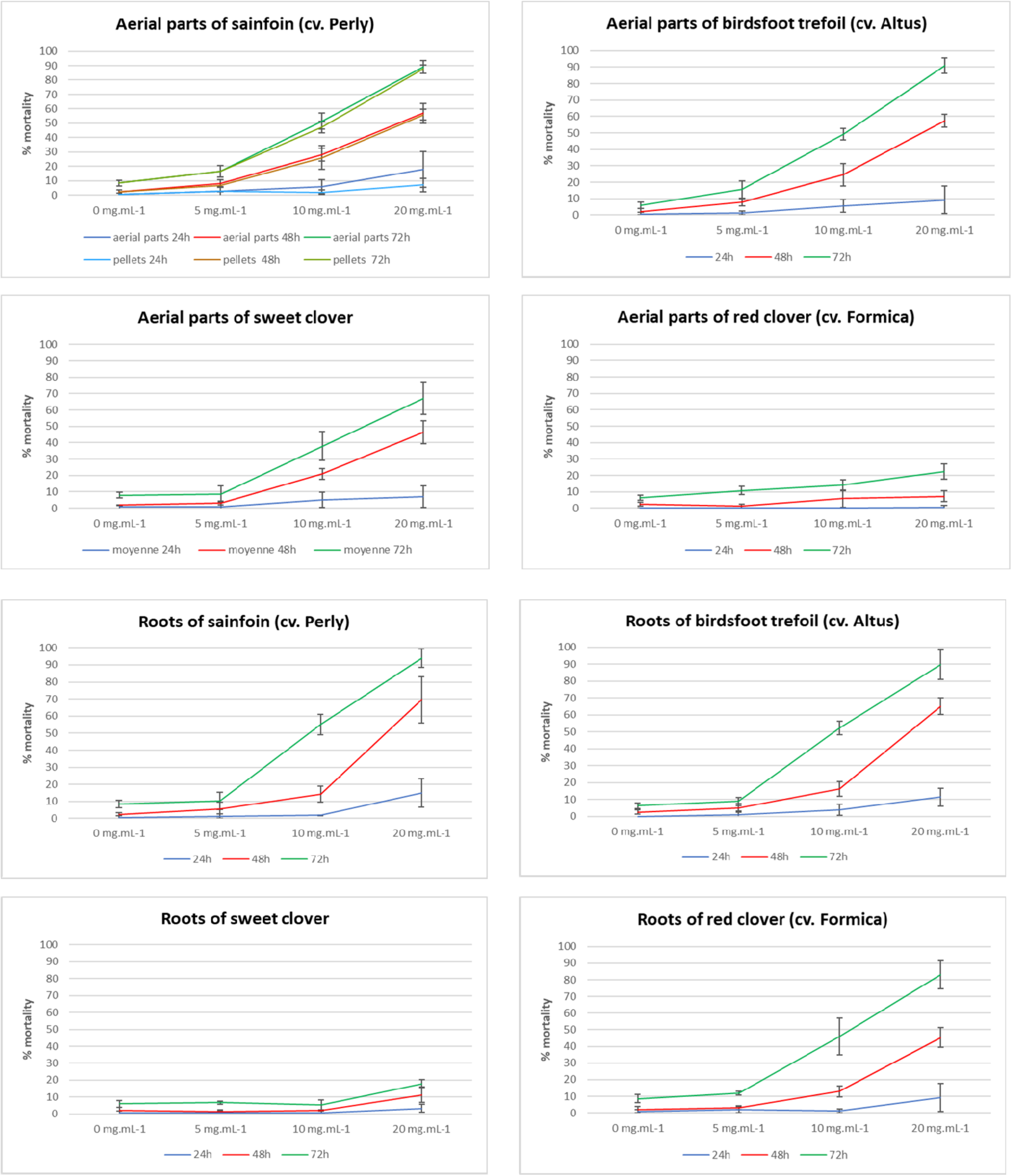

Supplementary Figure S1. Effect of aerial part and root extracts of Fabaceae on the mortality of *X. index in vitro*. Free *X. index* were incubated with indicated aerial part and root extracts. The extracts were concentrated at 0, 5, 10 or 20 g.L^{-1}. Bioassays were monitored for 72 hours. The results presented correspond to three independent repetitions and bars indicate lowest and highest % mortality in the three repetitions.

Supplementary Table S1. List of the 93 target molecules for metabolomic analyses. *m/z* : mass-to-charge ratio; RT: retention time; Glc: glucoside; GlcA: glucuronide; Rha: rhamnoside; Rut: rutinoside. Metabolites indicated with * were not detected in quantifiable amounts in any of the plant extracts. Compounds indicated in bold were confirmed with authentic standards. Compounds indicated in italics were identified from the published literature.

Molecule name	Abbreviation name	Molecule family	Molecular formula	Theoretical *m/z* positive	Experimental *m/z* positive	Error positive (ppm)	Theoretical *m/z* negative	Experimental *m/z* negative	Error negative (ppm)	RT (min)
**3-*O* -Caffeoylquinic acid**	3-O-Caffeoylquinic acid	Cinnamic acid	$C_{16}H_{18}O_9$	355.10235	355.10246	0.1	353.0878	353.0864	-4.1	2.46
**4-*O*- Caffeoylquinic acid**	4-O-Caffeoylquinic acid	Cinnamic acid	$C_{16}H_{18}O_9$	355.10235	355.10235	0.0	353.0878	353.0863	-4.3	2.62
**5-*O*- Caffeoylquinic acid**	5-O-Caffeoylquinic acid	Cinnamic acid	$C_{16}H_{18}O_9$	355.10235	355.10234	0.1	353.0878	353.0863	-4.3	2.10
**Caffeic acid**	Caffeic acid	Cinnamic acid	$C_9H_8O_4$	181.04953	181.04951	0.1	179.0350	179.0342	-4.6	3.39
**Cinnamic acid***	Cinnamic acid	Cinnamic acid	$C_9H_8O_2$	149.05971	149.05971	0.0	147.0452	147.0448	-2.3	7.91
Clovamide	Clovamide	Cinnamic acid	$C_{18}H_{17}NO_7$	360.10763	360.10763	-0.4	358.0917	358.0917	-4.1	3.76
**Ferulic acid**	Ferulic acid	Cinnamic acid	$C_{10}H_{10}O_4$	195.06518	195.06534	0.8	193.0506	193.0498	-4.5	5.27
**Glucosyl-o-coumaric acid**	Glc-o-coumaric acid	Cinnamic acid	$C_{15}H_{18}O_8$	327.10744	327.10768	0.7	325.0929	325.0915	-4.2	3.34
Glucosyl-p-coumaric acid	Glc-p-coumaric acid	Cinnamic acid	$C_{15}H_{17}O_8$	327.10744	327.10751	0.2	325.0929	325.0918	-3.4	4.28
**m-Coumaric acid**	m-Coumaric acid	Cinnamic acid	$C_9H_8O_3$	165.05462	165.05469	0.4	163.0401	163.0392	-5.0	6.18
**o-Coumaric acid**	o-Coumaric acid	Cinnamic acid	$C_9H_8O_3$	165.05462	165.05471	0.5	163.0401	163.0393	-4.8	6.98
**p-Coumaric cid**	p-Coumaric acid	Cinnamic acid	$C_9H_8O_3$	165.05462	165.05472	0.6	163.0401	163.0394	-3.8	4.79
Phaseolic acid	Phaseolic acid	Cinnamic acid	$C_{13}H_{12}O_8$	297.06049	297.06060	0.4	295.0459	295.0445	-5.0	4.04
**Sinapic acid**	Sinapic acid	Cinnamic acid	$C_{11}H_{12}O_5$	225.07575	225.07579	0.2	223.0612	223.0601	-4.9	5.04
**Catechin**	Catechin	Flavanol	$C_{15}H_{14}O_6$	291.08631	291.08631	0.0	289.0718	289.0707	-3.6	2.60
**Epicatechin**	Epicatechin	Flavanol	$C_{15}H_{14}O_6$	291.08631	291.08632	-0.3	289.0718	289.0708	-3.5	2.99
**Epicatechin gallate***	Epicatechin gallate	Flavanol	$C_{22}H_{18}O_{10}$	443.09727	443.09727	-0.1	441.0816	441.0816	-2.5	4.60
**Epigallocatechin**	Epigallocatechin	Flavanol	$C_{15}H_{14}O_7$	307.08122	307.08132	0.3	305.0667	305.0660	-2.2	2.25
**Epigallocatechin gallate***	Epigallocatechin gallate	Flavanol	$C_{22}H_{18}O_{11}$	459.09218	459.09218	-0.2	457.0776	457.0764	-2.7	3.10
**Gallocatechin**	Gallocatechin	Flavanol	$C_{15}H_{14}O_7$	307.08122	307.08129	0.2	305.0667	305.0661	-1.8	2.10
**Apigenin**	Apigenin	Flavonoid	$C_{15}H_{10}O_5$	271.06009	271.06015	0.2	269.0455	269.0448	-2.7	7.58
**Apigenin-7-*O* -glucoside**	Apigenin-7-O-Glc	Flavonoid	$C_{21}H_{20}O_{10}$	433.11292	433.11301	0.2	431.0984	431.0965	-4.3	5.83
**Apigenin-8-*C* -glucoside**	Apigenin-8-C-glc	Flavonoid	$C_{21}H_{20}O_{10}$	433.11292	433.11298	0.1	431.0984	431.0966	-4.1	3.33
**Baicalein**	Baicalein	Flavonoid	$C_{15}H_{10}O_5$	271.06009	271.05999	-0.4	269.0455	269.0445	-3.7	8.06

**Chrysoeriol**	Chrysoeriol	Flavonoid	$C_{16}H_{12}O_6$	301.07066	301.07068	0.1	299.0561	299.0550	-3.7	8.10
Chrysoeriol-4 '-O-beta-D-glucoside	Chrysoeriol-4 '-O-Glc	Flavonoid	$C_{22}H_{22}O_{11}$	463.12348	463.12346	-0.1	461.1089	461.1076	-2.9	5.80
Chrysoeriol-glucuronide	Chrysoeriol-GlcA	Flavonoid	$C_{22}H_{20}O_{12}$	477.10275	477.10301	0.5	475.0882	475.0872	-2.2	6.30
Chrysoeriol-malonyl-glucoside	Chrysoeriol-malonyl-Glc	Flavonoid	$C_{25}H_{24}O_{14}$	549.12388	549.12406	0.3	547.1093	547.1082	-2.0	6.89
Chrysoeriol-rutinoside	Chrysoeriol-Rut	Flavonoid	$C_{28}H3_2O_{15}$	609.18139	609.18145	0.1	607.1668	607.1654	-2.3	5.00
**Diosmetin-7-*O* -rutinoside***	Diosmetin-7-O-Rut	Flavonoid	$C_{28}H_{32}O_{15}$	609.18139	609.18137	-0.1	607.1668	607.1655	-2.2	5.15
**Formononetin**	Formononetin	Flavonoid	$C_{16}H_{12}O_4$	269.08083	269.08083	0.0	267.0663	267.0652	-4.1	8.95
**Formononetin-7-*O*- glucoside**	Formononetin-7-O-Glc	Flavonoid	$C_{22}H_{22}O_9$	431.13366	431.13370	0.1	429.1191	429.1175	-3.7	6.79
**Genistein**	Genistein	Flavonoid	$C_{15}H_{10}O_5$	271.06009	271.06018	0.3	269.0455	269.0444	-4.2	8.04
**Genistein-7-*O*- glucoside**	Genistein-7-O-Glc	Flavonoid	$C_{21}H_{20}O_{10}$	433.11292	433.11293	0.2	431.0984	431.0965	-4.3	4.77
**Isorhamnetin**	Isorhamnetin	Flavonoid	$C_{16}H_{12}O_7$	317.06557	317.06561	0.1	315.0510	315.0498	-3.8	8.53
**Isorhamnetin-3-*O* -rutinoside**	Isorhamnetin-3-O-Rut	Flavonoid	$C_{28}H_{32}O_{16}$	625.17631	625.17631	0.1	623.1618	623.1590	-4.4	4.90
Isorhamnetin-glucoside	Isorhamnetin-Glc	Flavonoid	$C_{22}H_{22}O_{12}$	479.11840	479.11859	0.4	477.1038	477.1019	-4.0	5.42
**Kaempferol**	Kaempferol	Flavonoid	$C_{15}H_{10}O_6$	287.05501	287.05506	0.2	285.0405	285.0392	-4.4	8.13
Kaempferol-3-(2",6"-di-O-alpha-L-rhamnosyl)-beta-D-glucoside	Kaempferol-Glc-Rha-Rha	Flavonoid	$C_{33}H_{40}O_{19}$	741.22366	741.22353	-0.2	739.2091	739.2062	-3.9	3.33
**Kaempferol-3-*O* -glucoside**	Kaempferol-3-O-Glc	Flavonoid	$C_{21}H_{20}O_{11}$	449.10783	449.10784	0.0	447.0933	447.0914	-4.2	5.52
**Kaempferol-3-*O* -rhamnoside**	Kaempferol-3-O-Rha	Flavonoid	$C_{21}H_{20}O_{10}$	433.11292	433.11295	0.1	431.0984	431.0965	-4.3	6.72
**kaempferol-3-*O* -rutinoside**	kaempferol-3-O-Rut	Flavonoid	$C_{27}H_{30}O_{15}$	595.16574	595.16584	0.2	593.1512	593.1488	-4.0	4.59
**Kaempferol-7-*O* -neohesperidoside**	Kaempferol-7-O-neohesp	Flavonoid	$C_{27}H_{30}O_{15}$	595.16574	595.16602	0.7	593.1512	593.1485	-4.5	5.11
Kaempferol-glucuronide	Kaempferol-GluA	Flavonoid	$C_{21}H_{18}O_{12}$	463.08710	463.08720	0.2	461.0725	461.0710	-3.4	5.75
Kaempferol-rhamnoyl-glucosyl-rhamoside_1	Kaempferol-Rha-Glc-Rha1	Flavonoid	$C_{33}H_{40}O_{19}$	741.22366	741.22372	0.1	739.2091	739.2066	-3.4	1.99
Kaempferol-rhamnoyl-glucosyl-rhamoside_2	Kaempferol-Rha-Glc-Rha2	Flavonoid	$C_{33}H_{40}O_{19}$	741.22366	741.22370	0.1	739.2091	739.2064	-3.6	2.75
**Luteolin**	Luteolin	Flavonoid	$C_{15}H_{10}O_6$	287.05501	287.05505	0.1	285.0405	285.0405	n.d.	7.50
**Luteolin-4'-*O*- glucoside**	Luteolin-4'-O-Glc	Flavonoid	$C_{21}H_{20}O_{11}$	449.10783	449.10789	0.2	447.0933	447.0914	-4.3	5.58
**Luteolin-7-*O* -glucoside**	Luteolin-7-O-Glc	Flavonoid	$C_{21}H_{20}O_{11}$	449.10783	449.10795	0.3	447.0933	447.0922	-2.5	4.37

**Luteolin-7-*O* -glucuronide***	Luteolin-7-O-GlcA	Flavonoid	$C_{21}H_{18}O_{12}$	463.08710	463.08712	0.0	461.0725	461.0713	-2.6	5.01
**Luteolin-7-*O* -rutinoside**	Luteolin-7-O-Rut	Flavonoid	$C_{27}H_{30}O_{15}$	595.16574	595.16601	0.4	593.1512	593.1491	-3.5	3.76
**Luteolin-8-*C* -glucoside**	Luteolin-8-C-Glc	Flavonoid	$C_{21}H_{20}O_{11}$	449.10783	449.10779	-0.1	447.0933	447.0918	-3.4	3.36
Luteolin-Rhamnosyl-Rhamnoside	Luteolin-Rha-Rha	Flavonoid	$C_{27}H_{30}O_{14}$	579.17083	579.17089	0.1	577.1563	577.1538	-4.4	4.40
**Myricetin**	Myricetin	Flavonoid	$C_{15}H_{10}O_{8}$	319.04484	319.04486	0.1	317.0303	317.0293	-3.3	6.84
**Myricetin-3-*O* -rhamnoside**	Myricetin-3-O-Rha	Flavonoid	$C_{21}H_{20}O_{12}$	465.10275	465.10295	0.4	463.0874	463.0863	-4.2	4.19
**Prunetin**	Prunetin	Flavonoid	$C_{16}H_{12}O_{5}$	285.07575	285.07575	-0.9	283.0612	283.0599	-4.5	9.71
**Quercetin**	Quercetin	Flavonoid	$C_{15}H_{10}O_{7}$	303.04993	303.04997	0.1	301.0343	301.0339	-4.7	7.36
Quercetin-3-(2",6"-di-O-alpha-L-rhamnosyl)-beta-D-glucoside	Quercetin-Glc-Rha-Rha	Flavonoid	$C_{33}H_{40}O_{20}$	757.21857	757.21863	0.1	755.2040	755.2008	-4.3	2.73
**Quercetin-3-*O*- glucoside**	Quercetin-3-O-Glc	Flavonoid	$C_{21}H_{20}O_{12}$	465.10275	465.10294	0.4	463.0882	463.0864	-3.8	4.35
**Quercetin-3-*O*- glucuronide**	Quercetin-3-O-GlcA	Flavonoid	$C_{21}H_{18}O_{13}$	479.08201	479.08215	0.3	477.0675	477.0665	-2.1	5.25
**Quercetin-3-*O*- rhamnoside**	Quercetin-3-O-Rha	Flavonoid	$C_{21}H_{20}O_{11}$	449.10783	449.10789	0.2	447.0933	447.0914	-4.2	5.70
**Quercetin-3-*O* -rutinoside**	Quercetin-3-O-Rut	Flavonoid	$C_{27}H_{30}O_{16}$	611.16066	611.16060	-0.1	609.1461	609.1433	-4.5	3.75
**Rhamnetin***	Rhamnetin	Flavonoid	$C_{16}H_{12}O_{7}$	317.06557	317.06560	0.1	315.0510	315.0501	-2.9	9.07
**Syringetin***	Syringetin	Flavonoid	$C_{17}H_{14}O_{8}$	347.07614	347.07607	-0.2	345.0616	345.0546	-3.2	8.33
**Syringetin-3-*O* -glucoside***	Syringetin-3-O-Glc	Flavonoid	$C_{23}H_{24}O_{13}$	509.12896	509.12888	-0.2	507.1144	507.1101	-2.2	5.53
**Ascorbic acid**	Ascorbic acid	Organic acid	$C_{6}H_{8}O_{6}$	177.03936	177.03936	0.0	175.0248	175.0240	-4.9	1.45
**Citramalic acid**	Citramalic acid	Organic acid	$C_{5}H_{8}O_{5}$	149.04444	149.04436	-0.6	147.0299	147.0292	-4.7	1.78
**Citric acid**	Citric acid	Organic acid	$C_{6}H_{8}O_{7}$	193.03427	193.03442	0.7	191.0197	191.0191	-3.3	1.76
**Ellagic acid***	Ellagic acid*	Organic acid	$C_{14}H_{6}O_{8}$	303.01354	303.01349	-0.2	300.9990	300.9977	-4.3	4.60
**Gallic acid**	Gallic acid	Organic acid	$C_{7}H_{6}O_{5}$	171.02879	171.02882	0.1	169.0142	169.0137	-3.2	1.80
**Linoleic acid***	Linoleic acid	Organic acid	$C_{18}H_{32}O_{2}$	281.24750	281.24741	-0.4	279.2330	279.2330	n.d.	11.27
**Linolenic acid**	Linolenic acid	Organic acid	$C_{18}H_{30}O_{2}$	279.23185	279.23183	-0.1	277.2173	277.2173	n.d.	10.05
**Malic acid**	Malic acid	Organic acid	$C_{4}H_{6}O_{5}$	135.02879	135.02879	n.d.	133.0142	133.0138	-3.4	1.58
**Quinic acid**	Quinic acid	Organic acid	$C_{7}H_{12}O_{6}$	193.07066	193.07065	-0.1	191.0561	191.0554	-3.7	1.42
**Succinic acid**	Succinic acid	Organic acid	$C_{4}H_{6}O_{4}$	119.03388	119.03388	n.d.	117.0193	117.0189	-3.7	1.65

**Arbutin***	Arbutin	Phenolics	$C_{12}H_{16}O_7$	273.09688	273.09687	0.0	271.0823	271.0811	-4.5	1.60
**Procyanidin A1***	Procyanidin A1	Proanthocyanidin	$C_{30}H_{24}O_{13}$	593.12896	593.12899	0.2	591.1144	591.1128	-2.7	3.97
**Procyanidin A2**	Procyanidin A2	Proanthocyanidin	$C_{30}H_{24}O_{12}$	577.13405	577.13412	0.1	575.1195	575.1174	-3.4	4.75
**Procyanidin B1**	Procyanidin B1	Proanthocyanidin	$C_{30}H_{26}O_{12}$	579.14970	579.14996	0.4	577.1351	577.1329	-3.9	2.04
**Procyanidin B2**	Procyanidin B2	Proanthocyanidin	$C_{30}H_{26}O_{12}$	579.14970	579.15002	0.5	577.1351	577.1330	-3.7	2.49
**Procyanidin C2**	Procyanidin C2	Proanthocyanidin	$C_{45}H_{38}O_{18}$	867.21309	867.21279	-0.3	865.1985	865.1957	-3.3	2.36
Prodelphinidin dimer1	Prodelphinidin dimer1	Proanthocyanidin	$C_{30}H_{26}O_{13}$	595.14461	595.14490	0.5	593.1301	593.1278	-3.8	1.80
Prodelphinidin dimer2	Prodelphinidin dimer2	Proanthocyanidin	$C_{30}H_{26}O_{14}$	611.13953	611.13986	0.5	609.1250	609.1283	-3.5	1.80
Prodelphinidin dimer3	Prodelphinidin dimer3	Proanthocyanidin	$C_{30}H_{26}O_{13}$	595.14461	595.14490	0.5	593.1301	593.1280	-3.5	2.15
Prodelphinidin dimer4	Prodelphinidin dimer4	Proanthocyanidin	$C_{30}H_{26}O_{13}$	595.14461	595.14472	0.2	593.1301	593.1281	-3.4	2.40
Prodelphinidin dimer5	Prodelphinidin dimer5	Proanthocyanidin	$C_{30}H_{26}O_{13}$	595.14461	595.14473	0.2	593.1301	593.1283	-2.9	3.00
Prodelphinidin tetramer	Prodelphinidin tetramer	Proanthocyanidin	$C_{60}H_{50}O_{27}$	1203.26122	1203.26152	0.0	1201.2467	1201.2430	-3.5	2.00
Prodelphinidin trimer1	Prodelphinidin trimer1	Proanthocyanidin	$C_{45}H_{36}O_{21}$	913.18210	913.18215	0.0	911.1676	911.1646	-3.4	1.80
Prodelphinidin trimer2	Prodelphinidin trimer2	Proanthocyanidin	$C_{45}H_{38}O_{21}$	915.19783	915.19782	0.0	913.1833	913.1800	-3.6	1.80
Prodelphinidin trimer3	Prodelphinidin trimer3	Proanthocyanidin	$C_{45}H_{38}O_{20}$	899.20291	899.20299	0.1	897.1884	897.1850	-3.8	2.00
Prodelphinidin trimer4	Prodelphinidin trimer4	Proanthocyanidin	$C_{45}H_{38}O_{19}$	883.20800	883.20782	-0.2	881.1935	881.1906	-3.2	2.00
Prodelphinidin trimer5	Prodelphinidin trimer5	Proanthocyanidin	$C_{45}H_{38}O_{19}$	883.20800	883.20778	-0.3	881.1935	881.1911	-2.7	2.25
Prodelphinidin trimer6	Prodelphinidin trimer6	Proanthocyanidin	$C_{45}H_{38}O_{19}$	883.20800	883.20751	-0.5	881.1935	881.1902	-3.7	2.50

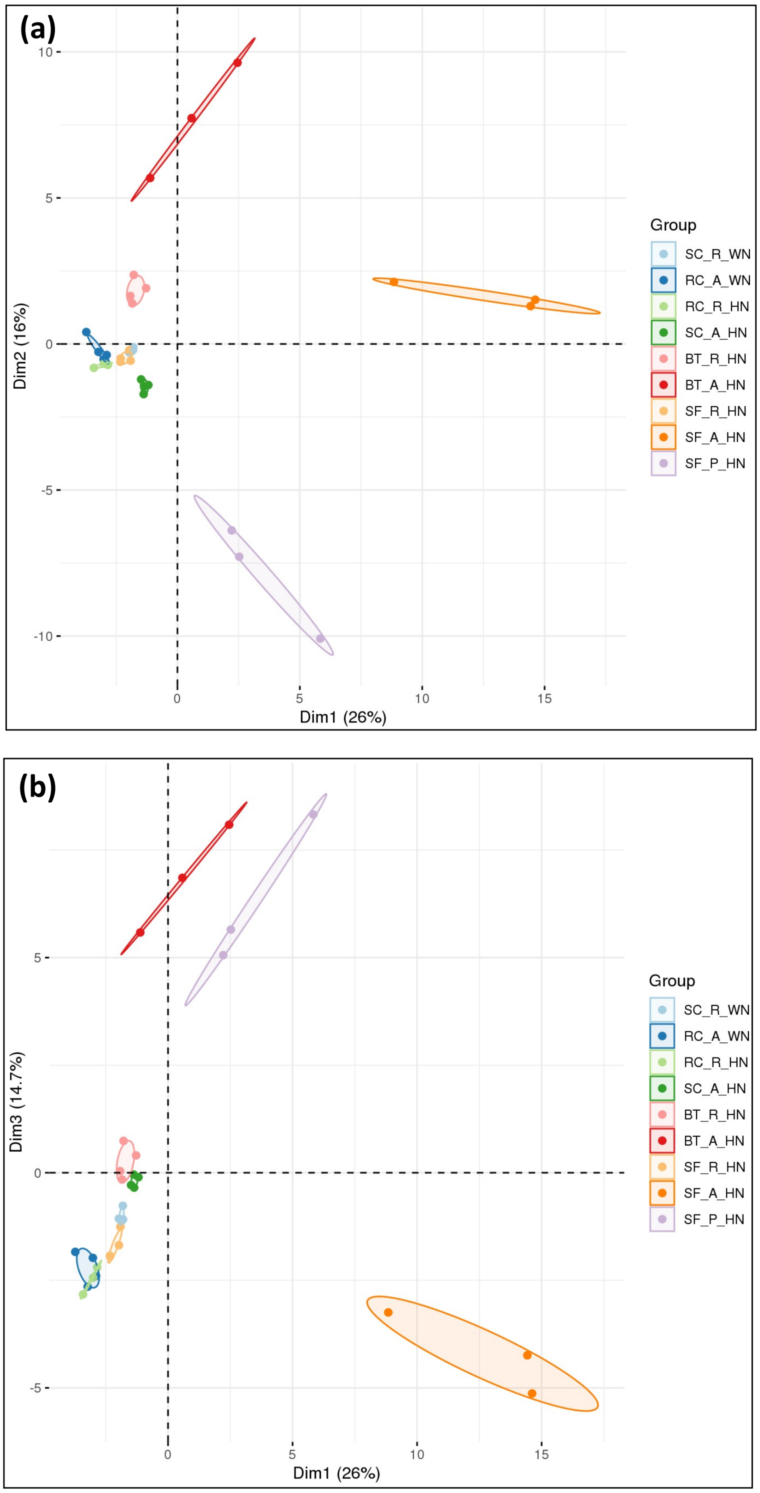

Supplementary Figure S2. Principal component analysis of metabolite contents in Fabaceae extracts. PCA was performed on relative amounts of all quantified compounds in the different Fabaceae extracts. Three or four replicates were performed for each analysis. The first two principal components explained 26% and 16% of the variance in the different groups of extracts, respectively (**a**). The third principal components explained 14,7% (**b**). SF: sainfoin, BT: birdsfoot trefoil, RC: red clover, SC: sweet clover. A: aerial parts, R: roots, P: pellets. HN: highly nematicidal; WN: weakly nematicidal extract.

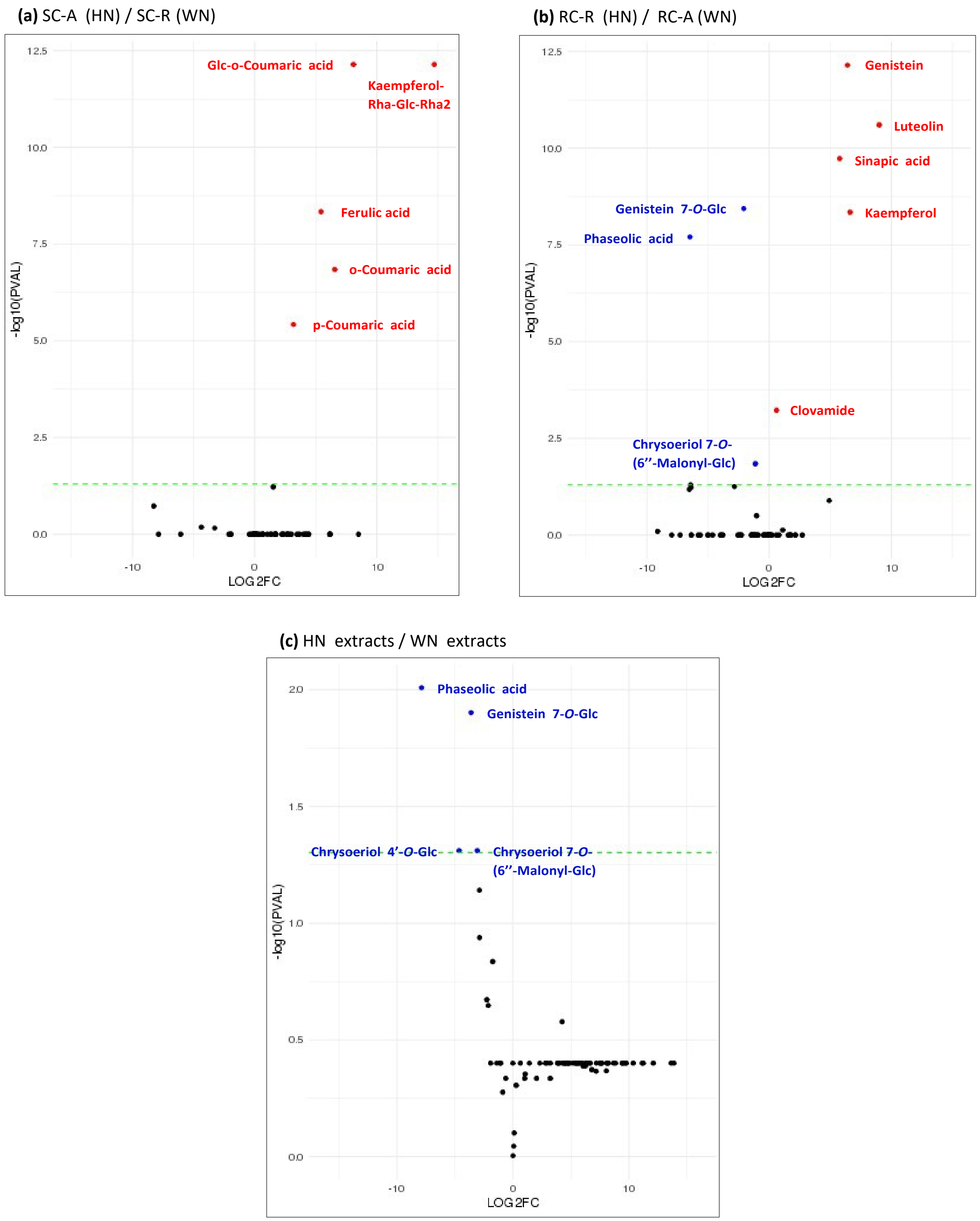

Supplementary Figure S3. Volcano plot analyses of differentially accumulated metabolites between highly nematicidal and weakly nematicidal Fabaceae extracts. Volcano plot analyses were used to identify significant differentially accumulated metabolites between the following plant extracts: (**a**) SC-A (HN) compared to SC-R (WN); (**b**) RC-R (HN) compared to RC-A (WN); (**c**) All HN Fabaceae extracts compared to all WN extracts; RC: red clover, SC: sweet clover. A: aerial parts, R: roots, HN: highly nematicidal, WN: weakly nematicidal. Red and blue colors indicate over-accumulated and under-accumulated metabolites, respectively. The green line indicates the significance threshold p value = 0.05.
